# Supramolecular self-assembly of a hybrid ‘hyalurosome’ for targeted photothermal therapy in non-small cell lung cancer

**DOI:** 10.1080/10717544.2020.1730521

**Published:** 2020-02-25

**Authors:** Haipeng Xu, Lin Dong, Zhang Bin, Huo Yansong, Lin Shaofeng, Liu Chang, Chen Chen, Wang Changli

**Affiliations:** a Department of Lung Cancer, Key Laboratory of Cancer Prevention and Therapy, Tianjin Medical University Cancer Institute and Hospital, National Clinical Research Center for Cancer, Tianjin’s Clinical Research Center for Cancer, Tianjin Lung Cancer Center, Tianjin, China;; b Department of Thoracic Oncology, Fujian Cancer Hospital, Fujian Medical University Cancer Hospital, Fuzhou, China;; c Department of Thoracic Surgery, Fujian Cancer Hospital, Fujian Medical University Cancer Hospital, Fuzhou, China

**Keywords:** Hyaluronate, calcium phosphate, indocyanine green, photothermal therapy, lung cancer

## Abstract

Despite the excellent efficacy and low toxicity of photoresponse therapy, which has attracted considerable attention for use in non-small cell lung cancer (NSCLC) therapy, unsatisfactory cellular permeability, and instability, both *in vitro* and *in vivo* have limited further clinical applications of indole cyanine photosensitizers. Here, we explore the supramolecular self-assembly of a ‘hyalurosome’ that is mediated by calcium phosphate nanonuclei. Through hyaluronate-mediated CD44 targeting, the constructed hyalurosome specifically delivers ICG into NSCLC cells and then induces severe hyperthermia accompanied by the generation of singlet oxygen upon photoirradiation. In contrast to the action of the native form, indocyanine green encapsulated in a hyalurosome showed significantly increased cellular endocytosis and inhibited cell proliferation both *in vitro* and *in vivo*. Our study indicated that this hyalurosome is a promising candidate for the targeted delivery of photosensitizers, which may be useful in NSCLC therapy.

## 1. Introduction

Lung cancer is one of the most common malignant tumors in the world, causing cancer deaths in one-quarter of males and one-fifth of females. Among these deadly cancers, non-small cell lung cancer (NSCLC) accounts for approximately 85% of all lung cancer cases and includes squamous cell carcinoma (SCC), adenoma carcinoma (AC), and large cell carcinoma (Singhal et al., 2005; Chen et al., 2014). Compared with small cell carcinoma, NSCLCs are relatively insensitive to cytotoxic chemotherapy and are highly prone to drug resistance (Reck et al., 2014). Currently, surgery is the preferred treatment for patients; however, only 25–30% of patients are ultimately suited for surgical resection with a curative intent (Chang, 2011; Schreiner et al., 2018). Therefore, new treatments are urgently needed to reduce the recurrence rate and mortality of the disease (Dong & Mumper, 2010).

In recent years, photoresponse therapy has attracted extensive attention due to its excellent efficacy and low toxicity and consists of photothermal therapy (PTT) and photodynamic therapy (PDT) (Jaque et al., 2014; van Straten et al., 2017). Under wavelength-specific laser irradiation, light energy can be converted into local hyperthermia (>42 °C) or reactive oxygen species (ROS) through the use of a series of photosensitizers, thus destroying tumor cells and neovascularization (Dang et al., 2017). In addition, these photosensitizers induce negligible toxicity in the absence of laser radiation; therefore, they do not cause systemic toxicity to other normal tissues that are not irradiated, which ensures accurate and effective specific killing effect on tumors (Moghissi, 2004).

Indocyanine green (ICG) is currently the only near-infrared (NIR) imaging reagent approved by the Food and Drug Administration (FDA) for clinical use, and it enables cardiac output and liver function measurements, ophthalmic angiography and sentinel lymph node detection (Schaafsma et al., 2011; Boni et al., 2015). ICG can be used in PTT or PDT (Jian et al., 2015) owing to the construction of a three-carbon cyanine dye with a NIR characteristic absorption peak, which can intensively absorb light energy and convert it into heat energy or produce singlet oxygen. However, the instability of ICG in aqueous solution and its rapid clearance rate in plasma (2–4 min of half-life) limit its application in diagnosis and treatment.

The inherent drawbacks of systemic administration have limited the application of ICG in cancer treatment. Moreover, the visible light decomposes easily, which makes storing it difficult and clinical applications challenging (Yan & Qiu, 2015). Among the possible considerations to address these issues, self-assembled nanoparticles provide a promising alternative since they can improve the physical and chemical stability of ICG (Yang et al., 2013; Zou et al., 2016). It is desirable to improve the treatment outcomes by minimizing the exposure of normal tissues to ICG while increasing the delivery of therapeutic concentrations of the drug to the tumor (Porcu et al., 2016).

Herein, a grafted polysaccharide was synthesized by conjugating the hydrophobic unit of dioleic acid to the carboxyl group of hydrophilic hyaluronic acid (DO-g-HA). We hypothesized that DO-g-HA serves as a lipid bilayer that envelopes an inorganic calcium phosphate (CaP) core through supramolecular self-assembly, which then targets delivery of ICG into tumor cells where it exerts PTT and PDT functions. As a kind of inorganic nanomaterial, CaP exhibits excellent inherent biocompatibility and biodegradability (Ridi et al., 2017). Upon endocytosis into cells, CaP undergoes dissolution in the acidic endosome and contributes to cargo release into the cytosol through endosome rupture (Satterlee & Huang, 2016). Unlike normal liposomes consisting of small phospholipid molecules, the hyalurosomes have an outer bilayer that is constructed with hyaluronate (HA) polymers. Moreover, the HA provides a targeting moiety for the hyalurosome such that it can specifically bind to NSCLC cells that overexpress the CD44 receptor (Kargi et al., 1997; Pirinen et al., 2001). The antitumor efficacy of this nanoplatform was investigated using NSCLC cell lines *in vitro* and a xenograft tumor model *in vivo*.

## 2. Experiments

### 2.1. Materials

Sodium HA with Mn 8.3–9.5 kDa was obtained from Bloomage Freda Biopharm Co., Ltd. (Jinan, China). Indocyanine green was obtained from Energy Chemical (Shanghai, China). N-Boc-3-amino-glycerin, 4-dimethylaminopyridine (DMAP), 1,2-dioleoyl-3-amino-propane (DOAP), and 1-ethyl-3-(3-(dimethylamino) propyl) carbodiimide (EDC) were obtained from Sigma-Aldrich (St. Louis, MO).

### 2.2. Cell culture

The A549 NSCLC cell line was obtained from the Cell Bank of the Chinese Academy of Sciences (Shanghai, China). All the cells were maintained in Dulbecco’s modified Eagle medium (DMEM) supplemented with 10% fetal bovine serum (FBS), 100 U/mL penicillin, and 100 µg/mL streptomycin (Invitrogen, Carlsbad, CA). Upon reaching 80–90% confluence, the cells were trypsinized and subcultured.

### 2.3. Synthesis of 1,2-dioleoyl grafted hyaluronic acid (DO-g-HA)

Oleic acid (10 mmol) was dissolved in 20 mL of dichloromethane, then N-boc-3-amino-glycerin (3.3 mmol), EDC (3 mmol), and DMAP (3 mmol) were added, and the mixture was stirred for 24 h at room temperature under nitrogen. The obtained Boc-protected DOAP was isolated and purified by silica column chromatography (petroleum ether/ethyl acetate ¼ 20:1). Subsequent deprotection was performed using a hydrochloride acid solution in 1,4-dioxane, which yielded 82% DOAP. For synthesis of the DO-g-HA, hyaluronic acid (HA, 5 μmol) was dissolved in formamide, and equal amounts (1.5 equivalent of HA) of EDC and NHS were added to activate the carboxyl group. Then, at various molar ratios, DOAP was introduced to HA, and the reaction mixture was agitated at room temperature in the dark. The product was dialyzed against distilled water (MWCO: 15 kDa, Viskase Companies Inc., Lombard, IL) successively to remove the reactant remnant after an additional 24-h incubation. The desired compound was obtained as a solid after lyophilization. The final products were generally obtained at a 70% yield. The ^1^H NMR spectra were recorded on a 300 MHz spectrometer at ambient temperature with CDCl_3_ as the solvent (Bruker AVACEAV-500, Fällanden, Switzerland).

### 2.4. Preparation of hyalurosomes with CaP as the core

First, oil-soluble CaP cores were synthesized via an inverse microemulsion system, as previously described (Shi et al., 2018). Briefly, 100 µL of 0.5 mM ICG was mixed with 300 µL of 2.5 M CaCl_2_ and then added to a 20-mL oil phase containing cyclohexane/IGEPAL CO-520 (70:30, v/v) to form a well-dispersed reverse microemulsion. Another aqueous solution, of 12.5 mM Na_2_HPO_4_, was also prepared and dispersed into the same oil phase. After stirring for 15 min separately, the two separate emulsions were immediately mixed together, and 2 μmol DO-g-HA was added. The mixed emulsion was continually stirred for another 30 min at room temperature before 40 mL of ethanol was added. Then, the mixture was subjected to ultracentrifugation at 10,000×*g* for 20 min to remove cyclohexane and the surfactants. The final pellets were washed twice with ethanol and then redispersed into chloroform for further modification. For the reconstitution of HA vesicles, the obtained CaP cores and another fraction of DO-g-HA were dissolved in 200 µL of THF/DMSO and added dropwise into 5 mL of water being stirred at room temperature. Then, the mixture was centrifuged at 2500×*g* for 10 min and 40,000×*g* for 20 min to remove the large aggregates and the solvent, respectively. Then, the resulting particles were washed again and redispersed into 2 mL of tricine buffer (5 mM, pH = 7.4). Further purification was performed using sucrose density gradient centrifugation to ensure accurate physicochemical characterization.

### 2.5. Characterization of the hyalurosomes

The surface morphology of the hyalurosomes was examined by transmission electron microscopy (TEM) with a JEM-1200EX microscope (Tokyo, Japan). Fourier transform infrared (FT-IR) spectra were recorded on a Bruker IFS55 spectrometer (Bruker, Fällanden, Switzerland) in the range of 4000–400 cm^−1^. The hydrodynamic diameter, polydispersity, and zeta potentials of the vesicles were measured by dynamic light scattering (DLS) analysis using a Zetasizer Nano ZS (Malvern Instruments, Malvern, UK). The amount of ICG entrapped within the hyalurosomes was determined indirectly, i.e. by measuring the amount of free ICG in the supernatant recovered after ultracentrifugation with UV spectrophotometry at 781 nm. The drug entrapment efficiency (E.E.%) was expressed as the percentage of the ICG difference between the initial amount of ICG and the free IC in the supernatant relative to the total amount used for the NP preparation.

### 2.6. *In vitro* drug release

The *in vitro* release profile of the hyalurosomes was determined in Tris buffer of various pH levels at 37 °C. At appropriate time intervals, aliquots of samples (100 μL) from the suspension fractions were subjected to ultracentrifugation at 40,000×*g* for 20 min. The amount of ICG released in the supernatant was evaluated by UV spectrophotometry analysis. The percentage of cumulative ICG released from the vesicles was calculated as the ratio of the amount of ICG released at time (*t*) to the initial amount used.

### 2.7. Quantitative determination of singlet oxygen

Singlet oxygen (^1^O_2_) generation from either free or hyalurosome-entrapped ICG was quantified using a fluorescent probe: singlet oxygen sensor green (SOSG). Briefly, an SOSG solution (2 μM) in degassed water was added to the free ICG or hyalurosome solution at a concentration equivalent to 10 μg/mL ICG. Then, the mixture was irradiated with an 808-nm laser at 2 W/cm^2^ for specific periods to produce different levels of O_2_. After irradiation, the fluorescence of SOSG upon 488-nm excitation was recorded at 525 nm.

### 2.8. Transfections and intracellular distribution

Cells were seeded on Lab-Tek chambered coverslips at a density of 2.5 × 10^5^ cells/mL. Upon reaching 50–60% confluence, the cells were transfected with hyalurosome-encapsulated ICG. After 1–8 h of incubation in Opti-MEM without FBS, the cells were placed in fresh serum-containing medium and incubated for an additional 24 h. Then, the cells were washed three times with cold HBSS and fixed with 4% paraformaldehyde. The cells were permeabilized for 10 min with 0.1% Triton X-100 in blocking solution containing 1% BSA in HBSS. The cells were then incubated with LysoTracker Green (1 μg/mL) for endosome/lysosome staining. Following counterstaining with DAPI, the coverslips were mounted on slides and examined with a confocal laser scanning microscope (Olympus FV1000-IX81, Tokyo, Japan).

### 2.9. Flow cytometry assay of uptake by cells

Cells were seeded in six-well plates at a density of 1 × 10^6^ cells/well. At 50–60% confluence, the cells were transfected with hyalurosome-encapsulated ICG. For the competition assay, which was used to assess receptor-mediated cellular endocytosis, a receptor-blocking control was set in parallel by incubating cells with serum-free medium containing excess free soluble HA polymer (10 mg/mL) for 1 h prior to transfection. After incubation at 37 °C for 8 h, the cells were washed three times with cold DPBS and treated with 0.25% trypsin–0.02% EDTA. The trypsinized cells were harvested by centrifugation and resuspended in DPBS. The intracellular fluorescence of the ICG was analyzed by using a BD FACSAria flow cytometer (Becton-Dickinson, Franklin Lakes, NJ, USA).

### 2.10. Cytotoxicity assay

Cells were seeded onto 96-well plates at a density of 5 × 10^3^ cells per well overnight. Then, the culture medium was replaced with fresh medium containing ICG-loaded hyalurosomes. After laser irradiation at a power density of 2 W/cm^2^ for 3 min, the medium was replaced with 100 μL of MTT solution (0.5 mg/mL in HBSS) for another 3 h of incubation at 37 °C. Then, the formazan that had formed was dissolved in 150 μL of DMSO, and the absorbance of each well was measured at 490 nm with a microplate reader. The relative cell viability is expressed as the difference in absorbance between the test sample and the negative control.

### 2.11. Tumor growth inhibition study

Female athymic nude mice aged 5–6 weeks were provided by the Laboratory Animal Center of Beijing Medical University (Beijing, China). All animal handling procedures were approved by the Tianjin Medical University Cancer Institute and Hospital Human Ethics Committee. The A549 NSCLC cell line was used to establish the mouse xenograft models. Each mouse was inoculated subcutaneously with 1 × 10^7^ cells. ICG was administered intravenously at 7.5 mg/kg when tumors reached an average volume of 50–70 mm^3^. The A549 xenograft tumor-bearing mice were randomly separated and placed into three groups (*n* = 5 per group): the ICG solution, ICG-loaded hyalurosome, and the PBS (control) groups. Subsequently, 24 h post injection, the tumors were subjected to photoirradiation for 5 min (1.0 W/cm^2^) at 785 nm or were not treated. All groups were treated every three days, and each group received four treatments. Tumor size and mouse body weight were measured during the experiment. The tumor volume (*V*) was calculated according to the formula *V*=*a*×*b*^2^/2, where *a* is the longest dimension of the tumor and *b* is the perpendicular diameter.

### 2.12. Statistical analysis

Significant differences between treatment groups were determined by one-way analysis of variance (ANOVA) among three or more groups or Student’s *t* test (for two groups). A value of *p*<.05 was considered significant.

## 3. Results and discussion

### 3.1. Synthesis and characterization of grafted DO-g-HA

**Figure 1.**
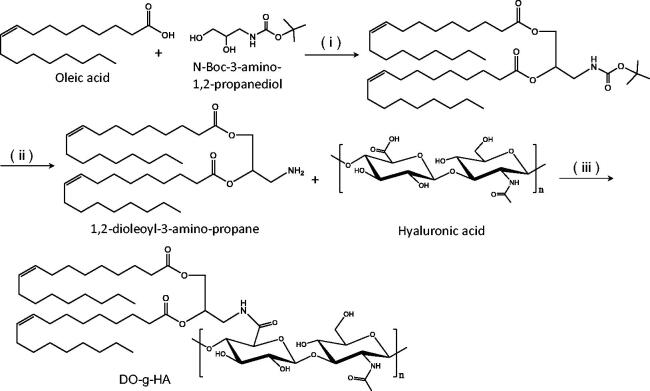
Synthetic route of DO-g-HA preparation. (i) EDC and DMAP; (ii) 4 M HCl/1,4-dioxane; (iii) EDC and NHS.

**Figure 2.**
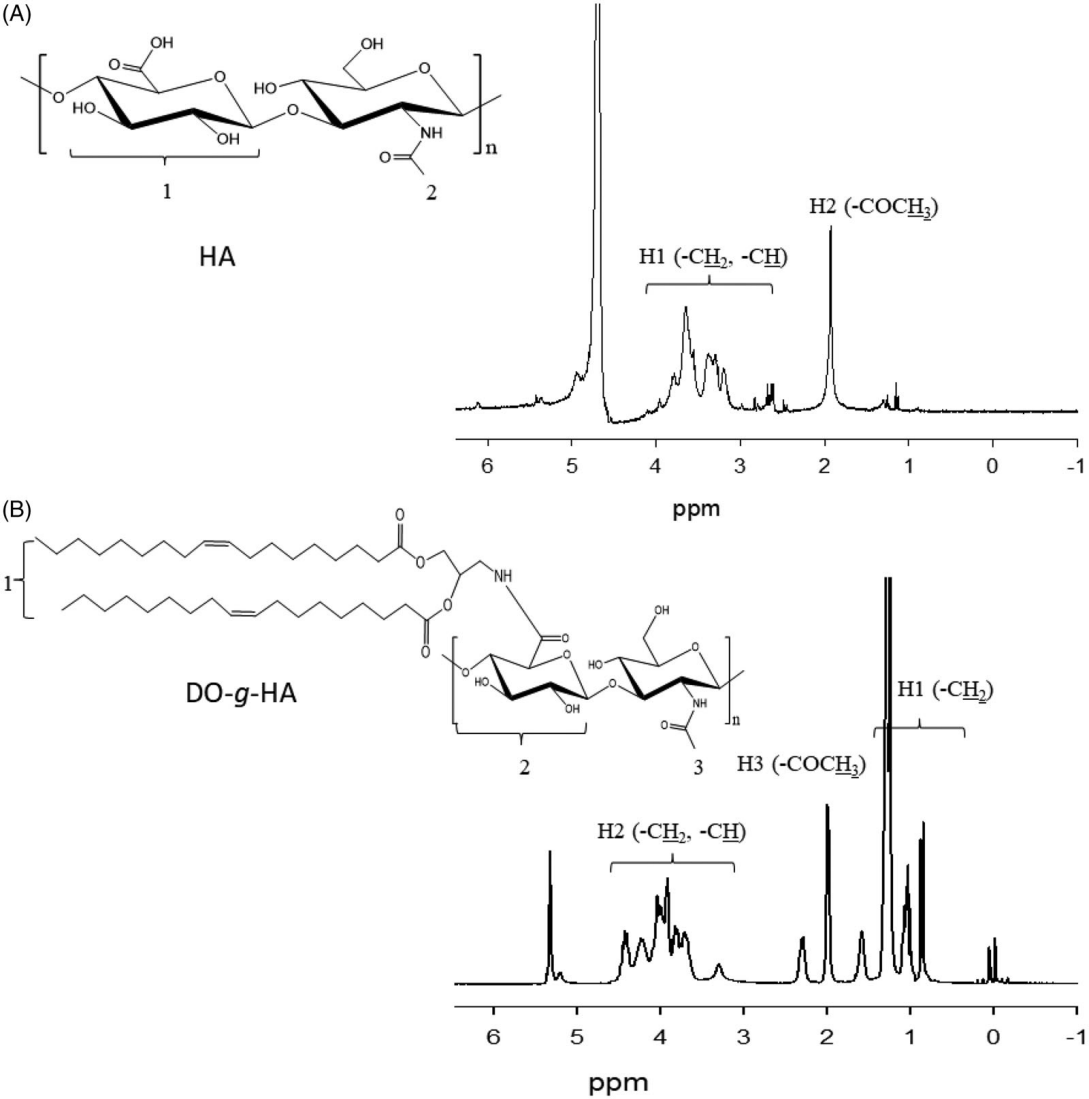
^1^H NMR spectrum of (A) hyaluronic acid (HA) and (B) DO-g-HA.

The polysaccharide, 1,2-dioleoyl hyaluronic acid graft (DO-g-HA), was synthesized by conjugating the hydrophobic unit of the dioleic acid to the carboxyl groups of the hydrophilic HA, which was catalyzed by EDC and DMAP (Figure 1). As shown in the results from the ^1^H NMR analysis, the proton peaks of DO-g-HA at 0.9–1.2 ppm can be attributed to the methyl and methylene groups in the long alkyl chain of the oleic acid moiety. In addition, multipeaks at 3.6–4.4 ppm represent the hydrogen of the pyranose of the HA. Both of these results proved that the oleic branches were successfully conjugated to the HA backbone. Moreover, new signals for the DO-g-HA graft at 2.0–2.5 ppm refer to the methane hydrogen of the amide bond (Figure 2), indicating that subsequent amino substitutions with carboxyl moieties were achieved.

### 3.2. FT-IR

**Figure 3.**
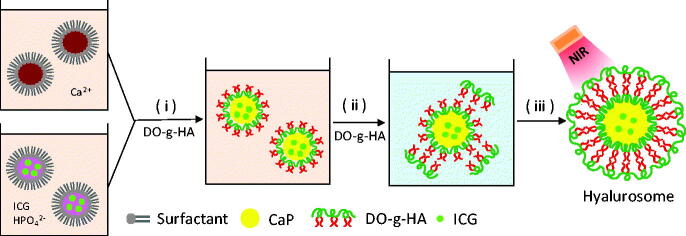
The outline for the preparation of the hyalurosomes. (i) Precipitation reaction between reverse microemulsions containing CaCl_2_ (a) and ICG/Na_2_HPO_4_ (b); (ii) removal of cyclohexane and the emulsifier by ethanol washing; and (iii) formation of CaP-supported hyalurosomes.

Figure 3 shows the FT-IR spectra of DO-g-HA, CaP, and the hyalurosomes.As observed in the FT-IR spectrum, two intensive IR absorption bands observed at 600–560 cm^−1^ and 1100–1000 cm^−1^ originated from the in-plane bending and asymmetrical stretching vibration of the PO_4_^3–^ group. In addition, the hyalurosome spectra revealed a broad stretching band in the region of 3200–3600 cm^−1^ and a sharp peak at 1620 cm^−1^; these represent the hydroxyl and carboxyl groups of the HA, respectively.

### 3.3. Preparation and characterization of the hyalurosomes

**Figure 4.**
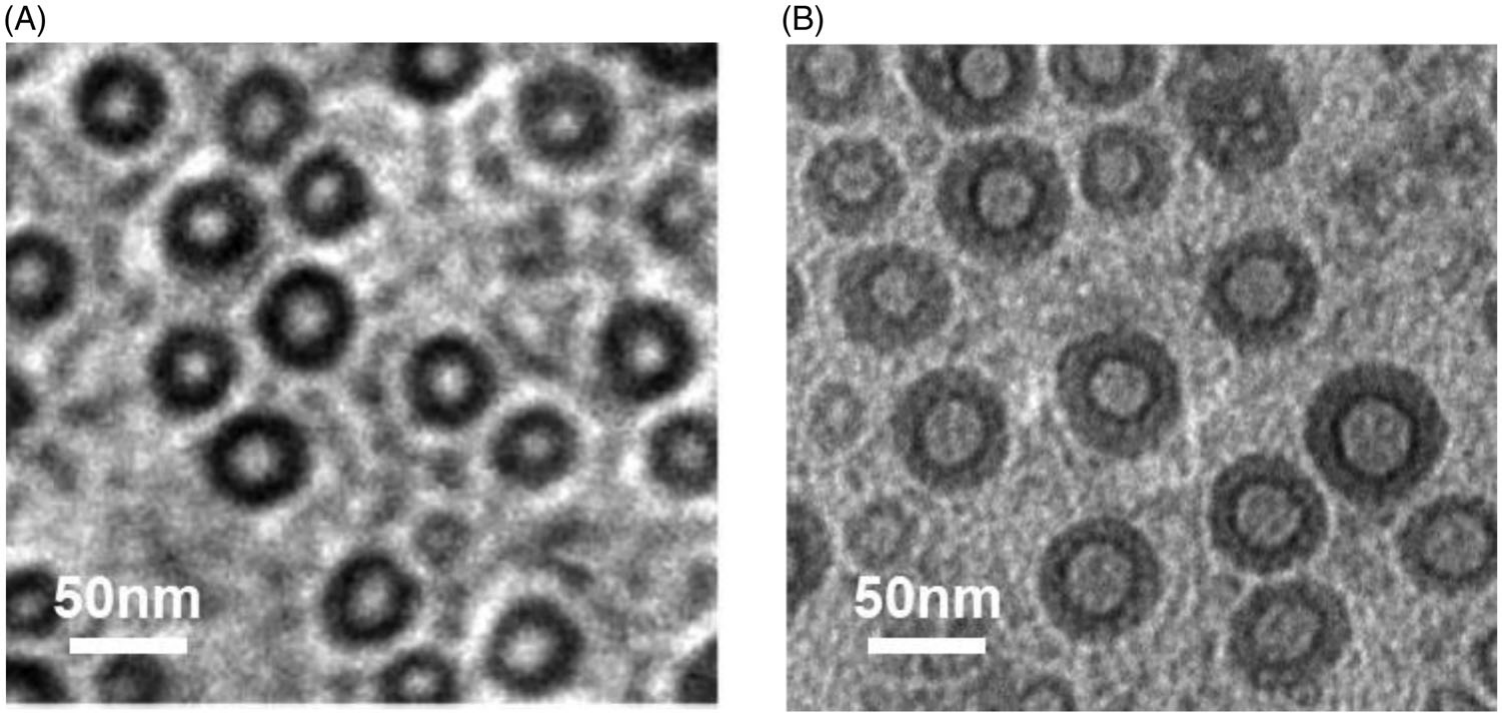
Typical TEM morphology of (A) CaP cores and (B) hyalurosomes.

A reverse microemulsion was used to create a two-phase interface system, in which the synthesis of CaP was performed on the cyclohexane/water interface (Figure 4). When the phosphate together with the DO-g-HA was mixed with the calcium in cyclohexane while being stirred at room temperature, interfacial emulsification spontaneously formed in the presence of reverse micelles. The DO-g-HA bilayer enveloping the CaP cores was prepared by simple thin-film hydration followed by extrusion method. In an aqueous solution, hydrophilic HA is attracted by ambient water to form an interface with the outer phase. The hydrophobic oleoyl groups in the two layers, comprising long hydrocarbon chains of the grafted HA, are repelled by the water and thus face each other to form a bilayer structure.

### 3.4. Characterization

**Figure 5.**
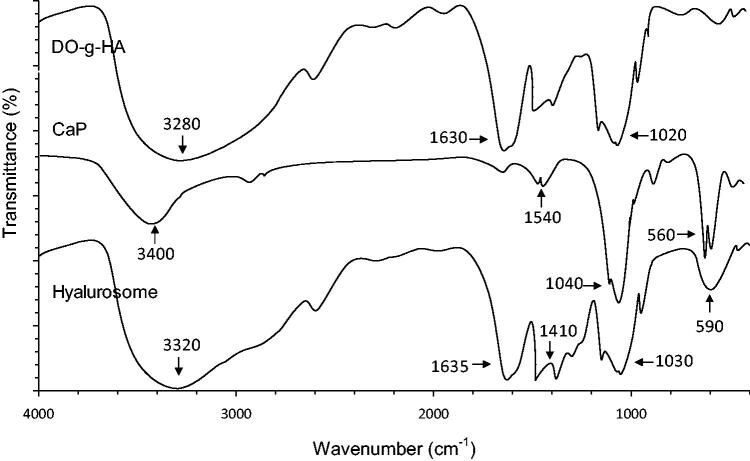
FT-IR spectra of DO-g-HA, CaP cores and hyalurosomes.

The TEM images of the CaP cores and the reconstituted hyalurosomes under typical experimental conditions are shown in Figure 5. The synthesized CaP core is quasi-spherical with a diameter of 40–50 nm. In the presence of DO-g-HA packages, an obvious coronal plane was observed around the surface of the CaP core. The hyalurosomes are highly monodispersed with a mean diameter in the range of 80–90 nm. In addition, the initial cargo input, based on the physicochemical properties of the hyalurosomes, such as the hydrodynamic diameter size (DH), polydispersity index (PDI), zeta potential, and drug encapsulation efficiency (E.E.%), is summarized in Table 1. With the increase of ICG added during the preparation of the CaP cores, both the particle size and PDI increased slightly, while the encapsulation efficiency decreased significantly.

**Table 1.** Characteristics of the hyalurosomes (*n* = 3).

Initial ICG input (%)	E.E.^a^ (%)	D.L.^b^ (%)	*D*_H_^c^ (nm)	PDI^d^
5	90.1 ± 2.6	4.6 ± 0.8	80.6 ± 3.7	0.19 ± 0.06
10	88.5 ± 3.4	7.5 ± 0.9	83.5 ± 2.8	0.24 ± 0.08
15	85.1 ± 2.9	8.8 ± 0.7	82.9 ± 3.1	0.28 ± 0.10
20	80.3 ± 1.5	9.2 ± 0.7	86.2 ± 4.5	0.32 ± 0.11

a Drug entrapment efficiency.

b Drug-loading capacity.

c Diameter of the hyalurosomes.

d Polydispersity index of the hyalurosomes..

### 3.5. *In vitro* drug release

**Figure 6.**
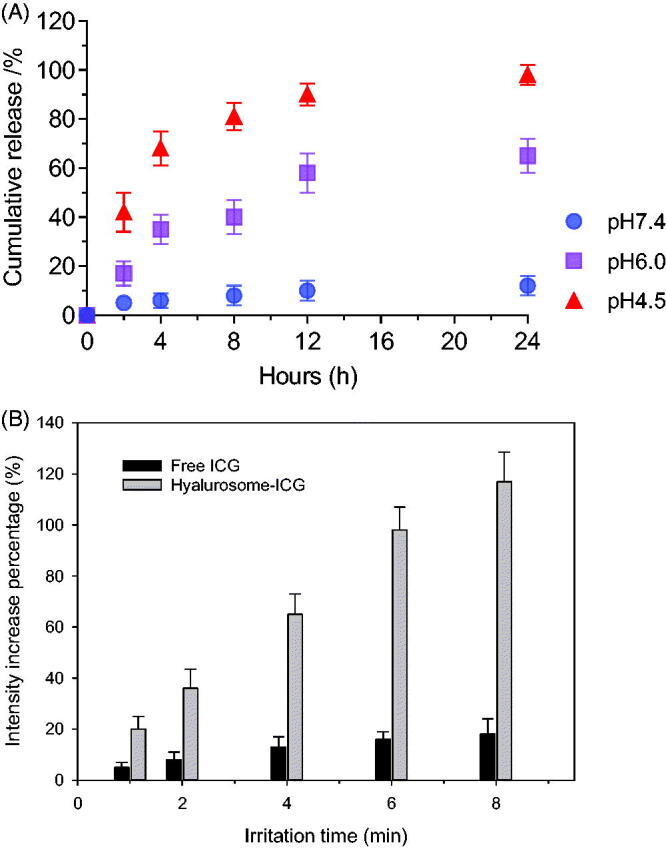
(A) *In vitro* release profiles of ICG from hyalurosomes in media at different pH levels. (B) The increase percentage of SOSG fluorescence intensities of the free and hyalurosome-encapsulated ICG.

*In vitro* release profiles of the ICG from the hyalurosomes were investigated in buffer solution at different pH levels. As shown in Figure 6(A), the dissolution rate was pH-dependent. When incubated in neutral medium, at pH 7.4, the hyalurosomes showed negligible cumulative release, which did not exceed 20% over the entire period. In contrast, particles were found to accelerate the release rate in medium between pH 6.0 and pH 5.0. In particular, more than 40% of the ICG was released during the first 2 h in medium at pH 4.5, and 80% of the cumulative drug release was achieved at 8 h. Compared with the 7.4 pH of blood and normal tissues, the extracellular environment of tumor tissues typically exhibits weakly acidity with an average pH of 6.8. In addition, the pH in the endosomes and lysosomes of the intracellular compartment further decreased, to 5.0–6.0 and 4.0–5.0, respectively (Ling et al., 2014). On the basis of the *in vitro* release behavior of the developed hyalurosomes, we can infer that the hyalurosomes did not dissociate immediately in the tumor tissue upon entering the cell. Once internalized into the cell, the nanoparticles dissociated in the acidic endosome and lysosome. The dissolved calcium and chloride ions induced an increase in osmotic pressure in the endosomes/lysosomes, causing them to swell and rupture, releasing the entrapped cargo into the cytoplasm.

### 3.6. Detection of singlet oxygen generation by hyalurosome-encapsulated ICG

Next, we quantitatively examined the ability of the hyalurosome particle to enhance singlet oxygen production by the ICG. As shown in Figure 6(B), the fluorescence intensity of SOSG gradually increased with prolongation NIR irradiation time, suggesting that ICG continuously produced singlet oxygen. Singlet oxygen has the lowest excited state of all molecular oxygen species and is widely considered to be the major cytotoxicity-inducing ROS generated during PDT. When optically excited, photosensitizers transfer energy to molecular oxygen to generate singlet oxygen, which can induce apoptosis or necrosis of cells in the targeted tissues by inducing oxidative stress. It was found that the SOSG fluorescence intensities of the hyalurosome-encapsulated ICG were 8.3-fold greater than those of free ICG. These results may be due to the high oxygen adsorption capacity of the CaP nanocores. Therefore, even though aqueous solutions may be saturated with oxygen before light irradiation, the hyalurosome-encapsulated ICG may carry a much greater amount of oxygen than free ICG dispersed aqueous solution.

### 3.7. Intracellular transport and distribution

**Figure 7.**
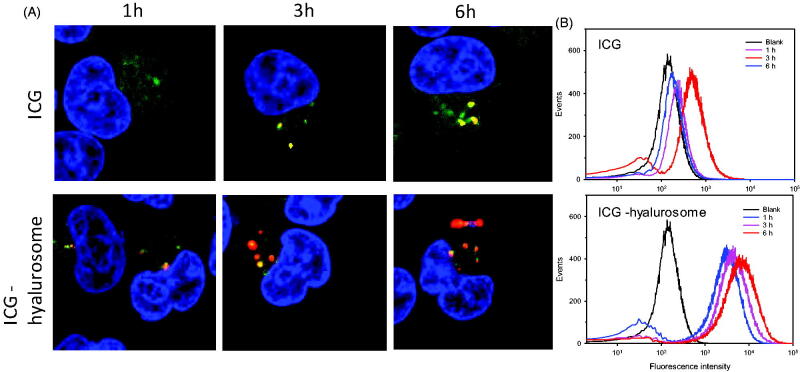
Intracellular trafficking of ICG during 6 h of incubation with tumor cells. (A) Subcellular localization of free and hyalurosome-enveloped ICG in A549 cells. (B) Quantification of the intracellular uptake of ICG, as indicated by flow cytometry analysis data.

As shown in Figure 7(A), the fluorescence of the hyalurosome-encapsulated ICG was observed within the cells after 1 h of incubation, indicating rapid hyalurosome internalization by the cells. In contrast, only weak fluorescence was observed in the cells treated with free ICG, even after 8 h of incubation. After internalization by the cell, ICG escape from the endosome/lysosome is a major threat to subsequent PTT efficacy in the cytoplasm. To evaluate the ability of the hyalurosomes to mediate ICG escape from the endosome/lysosome, the cells were incubated with free and hyalurosome-encapsulated ICG for various times at 37 °C. The distribution of endocytosed ICG in the cells was visualized by counterstaining the endosomes/lysosomes and nuclei with LysoTracker green and DAPI, respectively. As shown in Figure 7(A), the free ICG mainly localized to the endosome after 3 h of incubation, as indicated by the yellow pixels resulting from the colocalization of ICG with the LysoTracker green dye. In contrast, significant separation of the green and red fluorescence was observed in the cells that were incubated with hyalurosomes, suggesting the stronger endosomal escape efficiency of the hyalurosomes. After 6 h of incubation, both ICG from groups had escaped the endosome, but higher endosomal escape rates were found for the cells incubated with the hyalurosomes.

Cell populations were analyzed to quantify the cell internalization efficiency by flow cytometry. As shown in Figure 7(B), the offset of the average fluorescence intensity peak due to ICG status is slightly correlated with the inefficient cellular internalization of the free ICG, as observed with confocal microscopy, compared with that of the control group. In contrast, the average fluorescence intensity peak of the hyalurosome-encapsulated ICG was higher than that of the naked ICG, indicating improved cell internalization. Moreover, to confirm that the hyalurosomes are selectively internalized into cells by receptor-mediated endocytosis, a competition assay was performed by pretreating the cells with excess free soluble HA prior to transfection. The flow cytometry assay data showed a 60–75% reduction in the uptake of ICG in cells pretreated with excess HA (data not shown). These results indicate that the hyalurosomes were transported into cells mainly through the HA receptor-mediated endocytosis pathway.

### 3.8. *In vitro* evaluation of cytotoxicity

**Figure 8.**
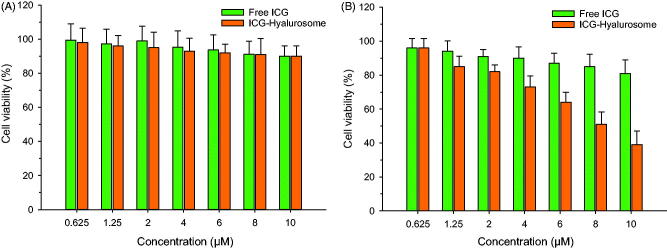
Cytotoxicity of the free and hyalurosome-encapsulated ICG without (A) or with (B) NIR exposure of the tumor cells (*n* = 6).

First, we estimated the cytotoxic effects of free and hyalurosome-encapsulated ICG on tumor cells at serial concentrations without NIR treatment. As shown in Figure 8(A), neither group exhibited significant cytotoxicity (*p*>.05), and more than 90% of the cells had survived after being treated at the tested concentrations. The results suggested that ICG was nontoxic to cells not subjected to NIR radiation. Next, we examined the cytotoxic effects of free and hyalurosome-encapsulated ICG upon NIR treatment. As shown in Figure 8(B), the free ICG had a weak inhibitory effect on tumor cells compared to the effect of ICG at the tested concentration range after NIR treatment. In contrast, hyalurosomes significantly enhanced the cytotoxicity of ICG to the tumor cells (IC_50_=8.1 ± 0.6 μM), and the cell survival rate, in particular, was decreased, by approximately 40%, compared with the survival rate in the non- radiation group after NIR radiation in cells with a high concentration of hyalurosome-encapsulated ICG (20 μM).

### 3.9. Tumor growth delay experiment

**Figure 9.**
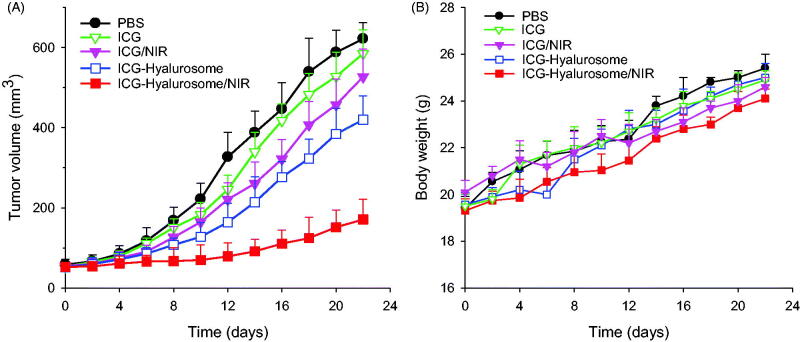
(A) Inhibition of tumor growth in the A549 xenografts by systemic administration of various ICG formulations (7.5 mg/kg). Data are expressed as the mean ± S.D., *p<.05 vs. the control; *n* = 5. (B) Weight changes in the A549 xenografted mice during treatment with various ICG formulations.

To verify the improved therapeutic effect of the hyalurosomes on ICG *in vivo*, various formulations of 7.5 mg/kg ICG were injected into the A549 cell xenograft mice every three days, and 24 h after hyalurosome-encapsulated ICG was administered, the tumors were irradiated at 1.0 W/cm^2^ for 5 min. All groups received four treatments, and tumor growth behavior was monitored for 21 days. As shown in Figure 9(A), intravenous administration of free ICG with or without NIR led to very poor tumor growth retardation, and no significant differences were observed compared with the with PBS control (*p*>.05). The results indicated that the NIR itself had a negligible influence on tumor growth in the absence of ICG. When delivered by hyalurosomes without subsequent NIR exposure, ICG showed a nonnegligible inhibitory effect on A549 tumor cell growth compared with the effect of the PBS control (*p*<.05), although the inhibitory effect on tumor growth was still limited. In contrast, ICG-loaded hyalurosomes significantly inhibited tumor growth after NIR exposure throughout the treatment period. These results suggest that the hyalurosomes effectively delivered a sufficient amount of ICG into the tumors, where it induced severe hyperthermia accompanied by the generation of singlet oxygen upon NIR exposure, and these effects jointly triggered lethal damage to the tumor cells (Sheng et al., 2014; Wan et al., 2014). Moreover, there was no significant change in mouse weight during the examination period (Figure 9(B)), indicating that both the hyalurosomes and NIR were biocompatible treatments and that the established dosing regimen can be applied for long-term cancer treatment.

## 4. Conclusions

In this study, the CaP nanonucleus-mediated supramolecular self-assembled hyalurosome was successfully prepared for improved delivery of ICG. Systematic design and modulation of a reverse microemulsion system, particle generation with satisfactory size and drug encapsulation efficiency of the hyalurosome were achieved with the proper control of the processing parameters. Moreover, an *in vitro* release study indicated that ICG was released from the hyalurosomes in a pH-dependent manner. The constructed hyalurosomes showed enhanced uptake by the cells and ICG-induced cytotoxicity in tumor cells both *in vitro* and *in vivo*. Therefore, we believe that this hyalurosome could be a potential carrier for the controlled release of ICG for effective antitumor activity.
